# The Effectiveness of an Empowerment Education Intervention for Substance Use Reduction among Inner-City Adolescents in Nigeria

**DOI:** 10.3390/ijerph20043731

**Published:** 2023-02-20

**Authors:** Hassana Shuaibu Ojonuba, Haliza Abdul Rahman, Zeinab Zaremohzzabieh, Nor Afiah Mohd Zulkefli

**Affiliations:** 1 Faculty of Medicine & Health Sciences, Universiti Putra Malaysia, Serdang 43400, Malaysia; 2 Institute for Social Science Studies, Universiti Putra Malaysia, Serdang 43400, Malaysia

**Keywords:** adolescents, inner city, empowerment education intervention, substance use reduction

## Abstract

(1) Background: Substance use among inner-city adolescents is at an alarming rate in Nigeria. Despite their high exposure to this risk, limited experimental tests have been conducted on prevention programs. (2) Methods: This study investigates the effectiveness of an empowerment education intervention in reducing the risk of substance use in Abuja’s inner-city adolescents. Random selection placed adolescents into intervention and control conditions, and assessment was conducted at baseline, post-test, and 3-months follow-up intervention. After pre-test, the intervention group engaged in an empowerment education intervention of 11 sessions. (3) Results: In a post-test of three months, results show significant and positive changes among adolescents in substance use, including a notable reduction in positive attitudes toward drugs. In other words, the results showed adolescents reported less depression and substance use as well as higher peer support, parental support, social competence, and self-esteem at post-test and 3-month follow-ups as compared to the pre-intervention period. In addition, at both post-test and the 3-month follow-up, the intervention group performed better than the control group on peer support, parental support, social competence, and self-esteem. (4) Conclusions: This study presents a new indication that the empowerment education intervention effectively reduces substance use among Nigeria’s inner-city adolescents.

## 1. Introduction

Substance use refers to a pattern of consuming or depending excessively on illegal or addictive substances, alcohol, and over-the-counter drugs, which is to the detriment of self and others [1]. Negative health implications include being vulnerable to infections due to a weakened immune system as well as various conditions of heart and liver failure or damage. It can also lead to negative behaviors, specifically paranoia, aggression, or hallucinations. As adolescents display impulsive and risk-taking behaviors [2], their vulnerability to substance use and use disorder is higher as they tend to choose instant gratification when by at large having a premature capacity to anticipate any negative consequences [3]. Characteristically, substance use has been reported to begin as young as 10 years old, and this increases steeply toward 19 years old [4]. The socio-geographical dwellings of adolescents play a role in substance use disorder; those living in lower-income neighborhoods in the city center, also known as inner cities, have reported higher risk [5,6].

Inner cities are characterized as an area of urban communities with high rates of impoverishment, violence, tenancy, single-head households, and limited access to the country’s political and social resources [7]. Inner cities also host a disproportional rate of minority groups. Therefore, interest in inner-city adolescents is growing, particularly in the area of health risks, including substance use. A one-year longitudinal study on American inner-city youths revealed that early alcohol consumption is associated with internalizing and externalizing behaviors [8]. Other studies found that not only does substance use impact inner-city adolescents negatively, but it also leaves significant and negative effects in the long term.

Moreover, the said problems are further amplified as this young population bears the brunt of socio-economic inequalities and specific access to quality intervention programs and services related to prevention [9,10]. To add to the concern, these urban poor adolescents are labeled as a high-risk group that is perceived as having deficits [11,12]. The current perception is that inner-city adolescents are not capable of following through with a drug and alcohol use prevention program, carrying the doubt as to whether they can be social change agents to break the chain of drug and alcohol use in their community systematically [13]. Therefore, intervention programs need to focus on empowerment [11,14]. Empowerment has been positioned as a mechanism of health promotion and wellness among youth [15]. Over the past 10 years, researchers have examined the role of empowerment on adolescent developmental outcomes including substance use [11]. Previous studies have shown a negative association between youth empowerment and drug and alcohol use [16]. Christens and Peterson [17] also found that youth with higher composite scores of intrapersonal psychological empowerment were both involved in more community activities and less likely to report substance-using behaviors. Thus, substance use can be reduced with such programs which also increase protective factors in adolescents, allowing them to affect social change in their community via an education empowerment model [14].

However, to our knowledge, no study has been conducted on an educational empowerment intervention for substance use reduction among inner-city adolescents. Therefore, this study aims to evaluate the effectiveness of an educational empowerment intervention to reduce substance use among inner-city adolescents in Abuja, Nigeria. We hypothesized that the intervention group would report less substance use compared with those from the control group. The risk-and-protective-factors approach has been used to guide substance use prevention research.

## 2. Literature Review

### 2.1. Substance Abuse among Inner-City Adolescents in Abuja, Nigeria

**Figure 1:**
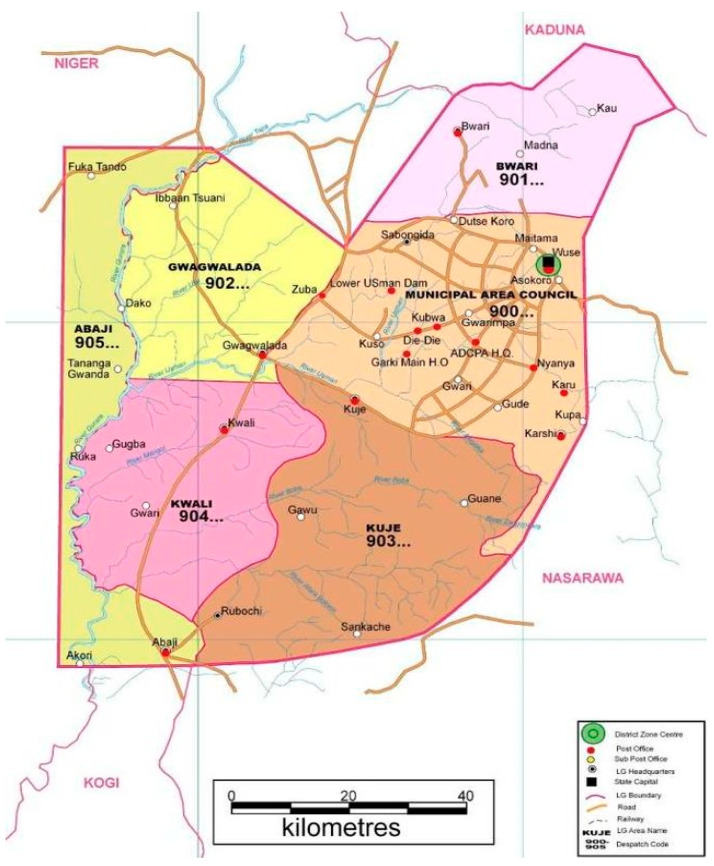
Map of Abuja, Nigeria [19].

Abuja is the capital city of Nigeria, and it is one of the fastest-growing capitals in sub-Saharan Africa (Figure 1). Located in central Nigeria, this city hosts approximately 3.5 million city dwellers. Similar to other developing countries, Nigeria’s urban centers have had persistent pressures from a growing population and a lack of employment opportunities. These mostly result in individuals turning to substance use and related activities. Recently, residential neighborhoods in Abuja have become a haven for drug dealers and/or users due to rapid immigrant population growth; slums emerged with the absence of urban planning to effectively consider geo-demographic changes [18].

A total of 1,108,350 adolescents make up 28% of Abuja’s population [20]. There are several peri-urban communities in Abuja, which have maintained their rural characteristics, located at the urban fringe. Due to the rapid development of infrastructure, these communities arose to become Nigeria’s economic and social capital. As the development occurred, the city saw a huge income gap being created between the predominantly wealthy occupants and low-income earners; the latter has been struggling with housing and access to other services available in this developed city. This led to the geo-segregation of low- and high-income groups where the former were forced to move to informal settlements and peri-urban communities [21]. In recent years, Abuja has been grappling with a growing drug use epidemic, specifically among its school-going adolescents [22]. Substance use helps them to temporarily forget about the harsh economic realities, namely unemployment and poverty. Drugs such as cocaine, crack, codeine, amphetamines, tramadol, marijuana, and LSD among others are now being abused by Abuja’s adolescent schoolers [23,24].

### 2.2. Risk and Protective Factors of Substance Use among Adolescents

Adolescence is a critical period for substance use initiation [25]. It is a phase marked by emotional, cognitive, biological, and neurological changes, which affect decision making and behavior [26]. Adolescents are also more likely to independently make decisions and place heavy significance on the thoughts and opinions of their peers. These changes predispose them to adopt risky behaviors, such as substance use [25]. Adolescent substance use can have severe and long-lasting effects. It can lead to delinquency, vandalism, poor academic performance, risky sexual behaviors, and addiction [27]. When initiated in adolescence, it can be sustained into adulthood and can be a predictor of health later in life [28,29]. These substances include the use of licit substances and illicit substances. Licit substances such as alcohol and tobacco have been associated with dependence, accidents, cancers, liver and lung diseases, and cardiovascular diseases [8]. Illicit substances such as cannabis and codeine have been associated with addiction, delinquency, poor physical health, and overdose [30].

The problem behavior theory [31] attempts to predict problem behavior, including substance use among adolescents, and it categorizes these factors into risk and protective factors. Risk factors are defined “as factors that increase the likelihood of involvement in risk behavior and lessen the likelihood of involvement in pro-social or health-enhancing behavior”; and protective factors are factors that “provide support for positive, pro-social behavior and development”. The theory adds that “protective factors interact with/moderate risk factors to reduce the likelihood of occurrence of risk behavior” [32]. According to the theory, increasing the protective factors and decreasing the risk factors reduce the likelihood of problem behavior among adolescents.

The theory posits that the protective and risk factors have a direct relation with risk behavior, while the protective factors have a moderating effect on the impact of the risk factor on risk behavior. Four types of risk and protective factors have been identified in the theory. The protective factors are: (1) Models protection—having role models in the adolescent’s social environment that engage in pro-social behaviors; (2) Control protection—having individual and social controls against engaging in risky behaviors; (3) Supports protection—having a social support system for positive and pro-social behavior; and (4) Behavior protection—experience with engagement in pro-social or protective behaviors. The risk factors identified are (1) Model risk—having models who engage in risk behaviors; (2) Opportunity risk—having opportunities to engage in risk behaviors; (3) Vulnerability risk—an individual’s vulnerability to engage in risk behaviors; and (4) Behavior risk—experience with engaging in risk behaviors [31]. Hence, it has been recommended that interventions should be holistic and preventive and should aim to increase the protective factors and decrease the risk factors. By increasing the protective factors, adolescents can build resilience, which helps them cope with life’s challenges and other risk factors and thus decreases their likelihood of using substances [31,33].

### 2.3. Freire’s Empowerment Education Model

As a framework, the empowerment education model effectively illustrates and explains the processes and outcomes of substance use prevention [11,17]. Interestingly, empowerment frameworks lay in contrast with community-based prevention approaches. The empowerment education model was developed based on Paul Freire’s philosophy which emphasized the need for critical consciousness through dialogue and collaborative work. He proposed three phases of learning: listening, dialogue and reflection, and action, which enables the students to analyze why, how, and who influences the problem and how they can address it. This empowering approach involves students identifying problems, determining the causes of those problems, and implementing strategies to address those problems, thus empowering them to change aspects of reality [34]. In the listening phase, themes and issues of the community are named with members determining priorities. In the dialogue and reflection phase, participants discuss with each other through a technique called problem-posing to understand the root causes of the issue. In the action phase, participants act toward trying to solve the issues identified by carrying out a form of social action or community service. This type of intervention has been reported to improve self-esteem, critical thinking, problem solving, community connectedness, and social support among the participants [35].

Matthews [36] proposes the application of this approach in health education due to its emphasis on raising awareness and promoting an active part in transformative action. The author states that this method is an empowerment approach that provides “learning which takes into account the complexity of health, and challenges learners to think critically about the social, cultural, economic, political and environmental determinants of health and the effect of these on individuals and populations” [36]. Despite these benefits, there is only weak evidence on the effect of these forms of interventions on adolescent substance use, especially in developing countries [37]. The existing programs are designed unsystematically, vary across time, and are hardly systematically evaluated [38]. Empowerment education interventions specifically are even scarcer, especially in developing countries such as Nigeria. Therefore, this research was aimed at developing an evidence-based empowerment education intervention and testing its effectiveness in reducing adolescent substance use among inner-city adolescents in Abuja, Nigeria.

## 3. Materials and Methods

The present study seeks to assess the empowerment education intervention on substance use as well as on behavioral outcomes associated with inner-city adolescent substance use in Abuja, Nigeria. This is a quasi-experimental study with a non-equivalent control group and a pre-post-test design, applying repeated measures [39].

### 3.1. Participants

The study participants were adolescents aged 12 to 17 years living in four peri-urban communities, which were randomly selected from a sampling frame of peri-urban communities in the Abuja Municipal Council Area. The sample size for the study was estimated using G-power. The prevalence of substance uses among adolescents who had received a substance use intervention vs. those who had not, as reported by Idowu et al., was used as a reference to calculate the sample size [40]. The initial sample size of 180 per group was obtained to detect a medium effect size (d = 0.5) between treatment conditions, with a 2-sided significance level of 0.05, and a power of 80%. After attributing a cluster design effect of 1.5 and a possible attrition rate of 10%, the sample size of 300 per group was obtained for this study. Adolescents between the age of 12 and 17 who used alcohol and/or other drugs, but did not meet the requirements for substance dependence, participated in the intervention. These adolescents experienced negative behavioral effects. In addition, all participants were enrolled in secondary schools and had basic reading and writing skills. This was necessary because participants were expected to fill out their questionnaires privately and independently to ensure anonymity. Adolescents were excluded if they were involved in any projects or programs related to life skills, peer education, or health education which could potentially impact the study’s outcome.

### 3.2. Intervention

Based on the empowerment education approach, the intervention was divided into three phases. Phase 1 is the listening phase where the adolescents act as co-learners with the health educators and contribute their experiences on substance use. Phase 2 is the dialogue and reflection phase where through discussions and group dialogue, they learn the root causes of adolescent substance use. Phase 3 is the action and reflection phase where the adolescents identify a substance use-related issue they wish to address in their communities and come up with creative strategies to address these issues. They develop and carry out a group service activity or project by implementing the project ideas and then reflect on the project they have carried out and how it has affected them.

In addition, formative research was carried out to obtain adolescents’ views on the intervention curriculum design and delivery and their understanding of specific components and characteristics of the intervention curriculum. The intervention group received weekly sessions every Saturday for 12 weeks according to Freire’s empowerment education model developed in these three phases. A total of 11 session activities were designed in this study, as outlined in Table 1. At the end of each session, each participant was given a homework activity which was discussed in the following session. The completion time per session was estimated at 14 min. Students in the control condition received regular, existing substance use therapies that were chosen and implemented by school district staff.

Participants completed questionnaires for the baseline prior to session 1, the 2-month follow-up at the time of session 8, and the 3-month follow-up one week after the completion of session 11 (Table 2). Within this approach, the therapist’s goals are to demystify knowledge, eliminate arguments based on authority, and generate a redefinition of hierarchies of power in clinical relationships, allowing the client to be active and exercise control of the process. Focus group discussion (FGD) was used to check the validity of this intervention. We can also claim that the therapists we used for the validity of this intervention in the FGD were all trained and certified by the Counseling Association of Nigeria (CASSON). This organization consists of qualified, authorized, and registered counselors.

### 3.3. Instruments

Adolescents first responded to questions about their demographics, including religious parents, having a peer role model [41], and substance use. This was followed by questions on protective factors, namely peer social support [42], self-efficacy [43], self-esteem [44], social competence [45], and parental support [46]. Participants also provided questions on risk factors, namely their attitude toward substance use [47], delinquent behavior [48], and depression [49] for conventical behavior. Across measurements, items include parental disapproval of substance, parental mentoring, friend’s disapproval of substance use, availability of substance at home, parental use of the substance, sibling use of substance, and friend’s use of substance. Participants also answered questions about various substance use during the past 30 days, specifically alcohol, cigarettes, and marijuana. Other drugs included inhalants, club drugs, steroids, cocaine, methamphetamines, prescription drugs, and heroin. Gifts were distributed to all participants for the completion of the pre-test, post-test, and 3-month follow-up, respectively. A pilot test of the instrument was conducted among 32 inner-city adolescents. Based on the pilot test findings, several adjustments were made to the instrument. We used Cronbach’s alpha to determine the internal reliability of the instruments, while expert judgment was applied to obtain its face and content validity.

### 3.4. Procedures

The project was granted ethical approval from Universiti Putra Malaysia Ethics Committee for Research Involving Human Subjects (JKEUPM), with the reference number JKEUPM-2020-432. The researcher recruited participants and obtained prior informed consent. As the study posed minimal risks to the participants, a waiver of parental permission was granted. The adolescents were informed that participation was entirely voluntary and that they had the right to consent or refuse. To participate, they provided personal details, such as the participant’s name, birth date, e-mail address(es), and mobile number (optional), onto a secure webpage. Random selection assigned participants to intervention or control conditions. Both study groups completed the program in the order of post-test followed by 3-month follow-up; they were reminded about these through various means of communication, namely postcards, e-mails, and telephone calls. Finally, online gifts were distributed to the participants for pre-test, post-test, and 3-month follow-up, respectively.

### 3.5. Data Analysis

For comparison analysis between the experimental group and a control group, Chi-square tests were employed to examine if there were significant differences in demographic characteristics, substance use, and risk and protective factors between the two groups. We also used covariance techniques to analyze and assess differences in risk and protective factors for substance use between the experimental and control group at 2- and 3-month follow-up, controlling for baseline measures. ANCOVA was performed to compare the experimental and control groups’ performance on each outcome at baseline and three months. In addition, ANCOVA was used to produce adjusted means, which represent the means of each group once the covariate(s) has been controlled. Logistic regression was used to examine dichotomous dependent variables. The number needed to treat (NNT) and absolute risk reduction (ARR) was also computed. To take into account multiple comparisons, we applied the Bonferroni adjustment.

## 4. Results

### Baseline and Attrition Analyses

Table 3 displays baseline participant characteristics for each experimental group. There were differences between the groups in terms of age, religion, and peer role models. In comparison, intervention adolescents (81%) reported a higher percentage of having a peer role model compared with control adolescents (68.7%). On any of the gender, religious parents, or substance use measures, there was no difference between the groups (*p* > 0.05). At 3-month follow-up, attrition analysis revealed that 85% of the sample (*n* = 254) had been effectively maintained, with equivalent numbers of missing adolescents evenly divided across the intervention and control groups. Baseline information was used to compare the number of participants in each group who discontinued the study after three months. On any of the substance use, risk and protective behavior, and dropout measures, there were no differences between the two groups. Additionally, there were no differences in any socio-demographic factors across groups of dropouts.

## 5. Outcome Analysis

### 5.1. Protective Factors

The results illustrated significant differences in four protective factors (i.e., peer support, parental support, social competence, and self-esteem) after 2-week post-intervention (Table 4). Experimental adolescents reported higher peer support than those in the control group in role-play situations related to drug use. At 2 weeks post-intervention, experimental adolescents received more support from their parents in reducing their substance use. The differences were significant at the 0.01 level for parental support. In addition, experimental adolescents reported higher social competency and self-esteem than control adolescents. At a 3-month follow-up, the participants also reported a slight improvement in social competency and self-esteem for reducing substance use; the differences were significant at the 0.01 level for both groups. However, at 2-week post-intervention and 3-month follow-up, differences in self-efficacy factor were non-significant.

### 5.2. Risk Factors

Meanwhile, experimental participants showed less risk for substance use compared to the control participants on measures of substance use and attitudes and depression after the 2-week post-intervention. The results showed that for adolescents who were exposed to the intervention, the delinquent behavior improved for the controls but not for the experimental group at a 3-month follow-up. On the other hand, no significant differences were found in the measures of delinquent behavior and substance attitude among experimental participants at a 3-month follow-up.

Table 5 presents the results of a logistic regression analysis of the impact of the intervention on the prevalence of substances used by adolescents, controlling for baseline use. At 2 months, no impact was reported for any and frequent cigarettes, shisha, alcohol, marijuana, cocaine, heroin, refnol, Indian hemp, inhalants, and any drug consumption during the past 30 days. The intervention, however, had a substantial impact on lowering respondents’ later usage of sleeping pills, codeine, and weed, according to the data. Furthermore, the results revealed statistically significant intervention effects for codeine, heroin, and alcohol at the final assessment, showing that adolescents in the experimental group were less likely to report regular use of codeine, heroin, and alcohol than those in the control group. The effect of recent frequent codeine use has diminished between 2-month and 3-month follow up, during the past 30 days.

The absolute risk reduction (ARR) is presented in Table 6 to show the decrease in the risk of the experimental group as compared to the control group. The ARR ranged from 0.954% for any alcohol use to sleeping pills. The number needed to treat (NNT) to prevent one additional event is also presented. The NNT is the inverse of the ARR and indicates the number of participants who need the intervention as compared to the control group.

The highest NNTs were found for any codeine and frequent codeine use (NNT = 24). In comparison, fewer individuals would need to be exposed to the intervention to prevent one event for marijuana and/or inhalant use (NNT = 5).

## 6. Discussion

The effects of substance use continue to be disproportionately felt by adolescents in Nigeria’s inner cities. Adolescents in inner cities frequently have less opportunity to access high-quality prevention intervention services due to a lack of resources. This is partly because there are not many financial options available, and because programs continue to see adolescents through a deficit lens rather than a framework focused on their strengths. Therefore, the educational empowerment intervention in this study can help to understand the protective variables in inner adolescent drug use prevention and discuss how to increase protective aspects and decrease risk factors. Overall, 150 adolescents participated in the intervention, whereas 148 adolescents made up the control group. The empowerment education approach was used to teach lessons during the intervention’s eleven weekly sessions. According to the published literature, this study is the first of its kind to develop and test the empowerment education intervention for inner adolescents. This study also adds to the growing body of research on educational empowerment programs’ effects on inner adolescents’ 30-day substance use prevention. Importantly, this study moves away from “risk-focused” research and prevention, emphasizing empowerment as a tool to engage adolescents in their community and as a kind of prevention. Researchers, prevention specialists, and social workers all need to think about how to give adolescents more agency and tap into the collective wisdom and resources that help foster community and adolescent partnerships for prevention.

Furthermore, these findings provide a preliminary understanding of the relationship between the intervention for educational empowerment and risk and protective factors and 30-day substance use. The findings indicate that the intervention appears to be moderated by pre-intervention adolescent substance use at both 2 and 3 months post-intervention. The intervention had effects on substance use. In other words, the results showed adolescents reported less depression and substance use, as well as higher peer support, parental support, social competence, and self-esteem at post-test and 3-month follow-ups as compared to the pre-intervention period. It seems that an educational program based on the empowerment model has been effective in improving the average scores of the aforementioned constructs and total empowerment. Bagheri [50] showed there has been a statistically significant change in the attitude toward substance use after the intervention as compared to the preintervention period. However, the results of this study suggest that delinquent behavior improved for the controls but not for the experimental group. This is not to say that carefully designed and implemented treatment programs cannot work to reduce delinquent behavior. The present results suggest, however, that intervention most likely to be implemented by the typical school system is less efficacious than a community-level change, at least not in the short run.

The outcomes of changes in protective and risk factors over 2- and 3-month follow-ups also revealed limited efficacy for the final three sessions of the intervention, which is known as the action and reflection stage. We asked adolescents to participate in a problem-posing and community action process at this stage. During these sessions, adolescents have expanded the educational dialogue and made visible their concern for drug abuse. However, some issues still need to be addressed in how empowerment strategies can have a better understanding of the action–reflection–action spiral that produces change. A model of empowerment education recognizes the need for external leadership in providing resources and expertise, but we did not hold any sessions to foster conversation and teamwork with community members (i.e., adolescents, parents, and teachers) so that shared visions and community-based leadership might emerge. This session can create a supportive community that can investigate failures and successes, and identify obstacles, yet still maintains motivation to address the root causes of drug use among adolescents.

Investigating the effects of educational empowerment intervention revealed that following the study, participants’ social competence significantly improved, indicating that education is a key factor in determining adolescents’ social competence. The considerable difference between self-esteem scores before and after the intervention suggested that education in this study had a significant effect on improving adolescents’ self-esteem. The difference between self-esteem scores before and after the intervention was significant, indicating that this study’s empowering education significantly improved the self-esteem of adolescents. According to research by Moshki [51], teaching people life skills improves their abilities (such as problem-solving, assertiveness, controlling anger, etc.) and gives them the power to put their knowledge, attitudes, and values into practice, leading to more positive motivation and behavior.

The results suggest clinicians utilize inner adolescents’ strengths and those of their communities as a tool for reducing risk behaviors such as drug use. This intervention puts into focus ways in which to reduce substance use among adolescents and consider adolescents within the scope of developing and planning prevention–intervention initiatives. This intervention provides experimental participants with opportunities for developing a critical awareness and engaging in opportunities for leadership and social change [13]. This intervention of educational empowerment emphasizes these developmental processes as key stress-buffering pathways in promoting adolescents’ wellness [13]. A major practice implication is a need for the education and training of adults to increase conscious power sharing, guiding adolescents and adults through critical reflection activities, and supporting the broad goal of critical social youth empowerment. It has been suggested that adolescents and adults would benefit from training that focuses on building youth–adult partnerships [52], although existing models of education empowerment have not provided details about how to support adults in developing this balance. Further practice-based research is needed to further understand how engagement in this intervention may influence adolescents differentially.

The study also found a substantial difference in drop-out rates comparing males and females. It probably occurred because of the withdrawal of male participants who withdrew when they became aware of the heavy tasks imposed by the intervention and is therefore unlikely linked to the development of substance use in the underlying male adolescents.

## 7. Limitation

This study is important for extending the adolescent empowerment and substance abuse prevention–intervention literature. The results of this study should be considered in light of several limitations. First, the study was limited to a group of Abuja adolescents in Nigeria, and it is unclear if the results can be generalized to other adolescent populations; hence, future research will be needed in other locations to establish generalizability. Second, we lacked information on variables such as that neighborhood environment, parental conflict, parental psychopathology, and substance use that may have been potentially crucial for the observed relationships [53]. The results also imply that racial/ethnic disparities and cultural appropriateness in adolescent substance use may require further focus. Simply implementing a prevention program designed for other racial/ethnic groups of adolescents may not be an effective strategy for Abuja adolescents. Future research might concentrate on family-centered solutions, academic components, and interventions with high school-aged Abuja students that are culturally relevant. Third, research is required to ascertain whether this intervention that considers racial/ethnic diversity may successfully prevent substance use in various groups. This study’s absence of measurements for the components driving substance use limited our knowledge of potential processes for change in these behaviors and constituted another flaw. To better understand the mechanisms and processes for transformation, particularly for drug use, more studies are required. Last but not least, this study was only able to do 2-week and 3-month follow-ups, but as we previously said, longer-term follow-ups might offer more details on the delayed effects of the short intervention on drug use among inner-city adolescents in Abuja, Nigeria.

## 8. Conclusions

Despite these limitations, this study contributes to the current literature that considers how educational empowerment contributes to the prevention of 30-day substance use among inner adolescents. The current study tried to construct an empowerment education intervention for Abuja adolescents based on Freire’s empowerment education model as a way to decrease or prevent substance use, depression, and delinquent behavior, and boost peer support, parental support, social competence, and self-esteem. The intervention resulted in significant changes in the risk and protective factors scores of Abuja adolescents in Nigeria. There were no statistically significant changes in the scores of the control group. This work is expected to contribute to a better understanding of risk and protective factors among inner adolescents as well as the intervention used to reduce substance use among them. Furthermore, it is hoped that this intervention will give academic professionals and education officials significant insight into addressing the problem of substance use among this population.

## Figures and Tables

**Table 1:** Intervention Activities.

Session	Topic	Summary of Activities	Technique
1	Introduction	Knowing Each Other Understanding the Project’s Goals Setting Rules and Consequences	Group Discussion and Dialogue
2	Building Trust and Teamwork	Animal Game Common Ground Unknot	Games and Group Activities
3	We Can All Be Social Actors	Understanding Social Action Why Social Action Adolescents and Social Action	Group Discussion and Presentations
4	Listening: Adolescent Substance Use I	Discussing Tobacco Use Discussing Alcohol Use	Problem Posing Using Images
5	Listening: Adolescent Substance Use II	Discussing Drug Use Substance Use and the Community	Problem Posing Using Images and Group Discussion
6	Dialogue: Why Adolescents Use Substances	Story Time Formulate Your Story	Problem Posing Using a Story and Group Activity
7	Dialogue: The Adolescent Substance Use Problem Tree	Understanding the Problem Tree Draw Your Problem Tree	Group Discussion and Presentation
8	Action: Selecting a Project Idea	Analyzing the Project Ideas Selecting a Project Idea	Group Discussion
9	Action: Planning the Project I	Plan, Plan, Plan	Group Dialogue
10	Action: Planning the Project II	Finalizing the Plans Visioning	Group Dialogue
11	Reflection	Questions and Answers	Group Dialogue

**Table 2:** The dropout rate at baseline, a 2-month, and a 3-month follow-up intervention.

Subject	Baseline	2-Month Follow-Up	3-Month Follow-Up
Male 100% (138)	0.6 (2)	31.15% (41)	0% (0)
Female 100% (162)	0% (0)	1.85 % (3)	0% (0)
Total 100% (300)	0.6 (2)	15.33% (46)	0% (0)

**Table 3:** Characteristics of Participants at Baseline by Group.

Characteristics	Intervention Group (*n* = 254)		Control Group (*n* = 272)			
	%	*n*	%	*n*	χ^2^	*p*-Value
Gender						
Male	37.9	95	30.6	113	1.307	0.253
Female	62.1	159	54.4	159		
Religion						
Muslim	54.4	162	49.6	148	4.04	0.044
Non-Muslim	45.6	136	50.3	150		
Age (SD/M)	13.89 (2.29)		13.15 (2.27)		3.89	0.005
Religious parents	87	213	77	261	0.093	0.76
Peer role model	68.7	103	81	119	5.94	0.015
Substance use	56.30	161	55	159	0.009	0.925

**Table 4:** Protective and Risk Factors in Substance Use: ANCOVA with Baseline Measures as Covariates.

	2-Month Follow-Up Adjusted Mean (SD)			3-Month Follow-Up Adjusted Mean (SD)		
	Control	Experimental	*n*	Control	Experimental	*n*
Protective Factors						
Peer support	19.564 (0.365)	21.126 (0.357) **	250	19.567 (0.457)	20.047 (0.441)	254
Self-efficacy	31.890 (0.572)	32.410 (0.552)	251	32.562 (0.622)	33.067 (0.595)	249
Parental support	19.216 (0.355)	19.357 (0.343) **	246	19.680 (0.330)	19.680 (0.341)	250
Social Competence	14.249 (0.454)	15.535 (0.433) *	254	15.348 (0.370)	15.990 (0.450) **	246
Self-esteem	22.316 (0.367)	22.326 (0.388) *	244	22.303 (0.375)	22.362 (0.387) **	249
Risk Factors						
Positive attitude toward substance abuse	4.359 (0.081)	4.331 (0.078) *	254	4.231 (0.090)	4.231 (0.094)	254
Delinquent behavior	6.428 (0.758)	3.931 (0.729)	254	4.295 (1.00)	4.073 (1.051) **	249
Depression	6.461 (0.332)	5.483 (0.343) *	246	6.249 (0.380)	6.128 (0.398)	251

* *p* < 0.05; ** *p* < 0.01.

**Table 5:** Logistic regression analysis comparing substance vs. abstinence across experimental and control subjects, controlling for baseline use.

	Cigarettes	Snuff or Shisha	Alcohol	Marijuana	Cocaine	Heroin	Refnol and Blue Boy	Indian Hemp	Sleeping Pills	Codeine	Weed	Inhalants	Any Drugs
	b(SE)	b(SE)	b(SE)	b(SE)	b(SE)	b(SE)	b(SE)	b(SE)	b(SE)	b(SE)	b(SE)	b(SE)	b(SE)
2-month follow-up													
Control Group	2.708 (1.032) **	2.566 (0.70)	1.83 (0.538) **	2.56 (0.32)	1.63 (0.39) **	1.71 (0.45) **	2.398 (1.044) **	2.079 (1.061)	2.94 (1.026)	2.058 (0.906) *	2.197 (0.94) *	2.079 (0.75)	1.12 (1.008) *
Experimental Group	−0.733 (1.052)	−0.6 (0.38)	1.14 (0.62)	−1.16 (0.38)	−0.16 (0.40)	−0.15 (0.43)	−0.169 (1.066)	1.8 (1.15)	−0.719 (1.04) **	−2.015 (0.752) **	−2.155 (0.745) **	1.417 (0.842)	−0.826 (1.7)
3-month follow-up													
Control Group	2.44 (1.65) **	2.5 (0.95)	2.833 (1.29)	2.56 (0.82)	1.45 (0.39) **	1.65 (0.50) **	2.944 (1.02) **	2.56 (1.96)	2.39 (1.044) **	2.56 (0.83)	2.56 (0.42)	2.56 (1.066)	2.56 (0.71)
Experimental Group	−0.66 (0.56)	−0.6 (0.84)	−0.047 (1.06) **	−1.85 (0.84)	−0.56 (0.39)	−1.06 (0.49) *	−0.719 (1.04)	−1.85 (1.93)	−0.169 (1.066)	−1.85 (0.58) **	−1.66 (0.23)	−1.25 (1.025)	−1.169 (0.49)

Note. The odds ratio is statistically significant. *p* < 0.008 qualifies as statistically significant. To take into consideration the Bonferroni adjustment, CI was adjusted to 99.2%. * *p* < 0.05; ** *p* < 0.01.

**Table 6:** Deviations from Baseline at 3-Month Follow-up, Bonferroni Corrected.

	Baseline Prevalence		3-Month Follow-Up
During the Past 30 Days	Control	Experimental	ARR ^b^ (NNT ^c^)
	%(*n*) ^a^	%(*n*)	%(*n*)
Cigarettes	4.2 (6)	2.3 (1)	0.957 (6)
Snuff or shisha	5 (13)	2.7 (7)	2.971 (13)
Alcohol	6.9 (18)	5.8 (15)	0.954 (14)
Marijuana	2.3 (6)	1.9 (5)	0.976 (5)
Cocaine	2.3 (6)	0.4 (1)	1.2 (6)
Heroin	2.5 (1)	0.4 (1)	1.4 (13)
Refnol and blue boy	3.990 (10)	1.2 (3)	0.961 (10)
Indian hemp	14.96 (38)	9.44 (24)	5.5 (18)
Sleeping pills	20.86 (53)	14.173 (36)	11.37 (14)
Codeine	18.11 (46)	9.84 (25)	4.1 (24)
Weed	15.35 (19)	8.26 (16)	7.09 (14)
Inhalants	10.23 (26)	5.90 (15)	1.18 (5)
Lifetime			
Subjects using any substance	4.6 (12)	3.01 (8)	0.952 (8)

Note. ^a^ n represents the number of participants after 3-month intervention, ^b^ Absolute risk reduction, ^c^ Number needed to treat.

## Data Availability

The raw data supporting the conclusion of this article will be made available by the authors, without undue reservation.
